# Estimation of age using pulp/tooth area ratio using orthopantomographs and intra-oral periapical radiographs

**DOI:** 10.6026/9732063002002040

**Published:** 2024-12-31

**Authors:** Monika Mehru, Rohit Sharma, Vipin Kumar, Himani Hazara, Shubhangi Joshi, Kumar Gaurav Chhabra, Amit Reche

**Affiliations:** 1 Department of Oral Pathology & Microbiology, NIMS Dental College & Hospital, Jaipur, Rajasthan, India; 2 Department of Public Health Dentistry, Sharad Pawar Dental College, DMIHER, Sawangi Meghe, Wardha, Maharashtra, India

**Keywords:** Age determination, forensic science, pulp, orthopantomography, radiography

## Abstract

Teeth are crucial for assessing age, as they remain intact even after death in forensic dentistry. Various regression models, such as polynomial regression, new robust regression equations, multiple linear regression models and partial least squares linear regression, are used to determine age. The pulp/tooth area ratio (AR) of maxillary canines is a direct link between age and the subject's age, making multiple regressions the most reliable method. Teeth hardness and invariant changes in their structure also strengthen teeth's applicability in estimating age. These statistical methods are suitable for both forensic and clinical applications, making them suitable for both forensic and clinical applications.

## Background:

The field of forensic medicine is the basis for many significant purposes such as criminal cases and trials, determination of cause of death and identification of deceased in the case of mass disasters or wars. Among the vast amount of techniques and methods employed in the forensic sciences, forensic odontology is regarded as unique since the source of identification in the given field is the teeth and these are the only elements that remain unchanged and recognizable even if other methods of identification are beyond application [1]. The process of locating ante mortem and postmortem records after the demise of a person is called forensic odontology began through Oscar Amoedo early in the 20th century [2]. It was in Amoede's doctoral dissertation, "L' Art Dentaire en Medecine Legale," where dental findings made extraordinary precedents to the legal process. His research and subsequent accomplishments set the foundations for the identification of dental remains as an important parameter in aspects such as the identification of individuals and age estimation [3]. The tissues of the oral cavity are made mostly of cement, enamel and dentin; these hard structures have a much longer lifespan than most other body tissues. This resilience is very useful in forensic situations where a deceased person's body may have decomposed due to fire, putrefaction, or other circumstances that prevent outside identification of the deceased person. Therefore dental remains are often the vital kind of evidence when an attempt is made to identify an individual and at some instances, the only conclusive way is done [4, 5]. Therefore, it can be said that dental age estimation plays a critical role in forensic odontology. In situations where identification of age by visual method or by assessing the skeletal features may not be enough or is not possible, then teeth may be used as a more accurate method [6]. It can entail the observation of developmental milestones, changes in morphology and radio graphical features of teeth in order to predict an individual's age at different times in his/her life cycle. This information proves very helpful in courts, especially when trying to seek the legal culpability of a certain person, reaffirming an age alibi, or recognizing victims of age-associated crimes [7]. The development of the dental age estimation approach is characterized by both progress in methods and technologies used. Now, from simple visual assessment and conventional radiography up to histological examination and digital photographic techniques, forensic odontologists are equipped with the instruments that allow them to provide an accurate estimation of age based on dental remains [8]. Of all the methods, intraoral periapical radiographs (IOPA) and orthopantomography (OPG) should be regarded as the most effective ones due to the opportunity to present detailed images of dental constructions to make further precise measurements and use the most important value-pulp/tooth area ratio in the context of age assessment [9].

This work seeks to compare the effectiveness of two techniques, IOPA and OPG, in estimation of teeth age using pulp/tooth area ratio. In doing so, this research aims at expanding the knowledge of the accuracy, reliability and practical utility of the above-mentioned imaging techniques in the hope of improving the quality of the forensic age estimation methods available today [10]. Lastly, improving the capacity of our team to determine age from dental angle positions does not only promote adequate investigation in forensic science but also provides justice and clarity in legal issues that greatly depend on age. The present study comparing two modes of imaging will try to establish which is more conducive to accurate age estimation, which will help in boosting forensic odontology. Through this evaluation of the methods, this study aims at contributing to the improvement of the percentage accuracy of age estimation from dental remains with the view of ensuring improved reliability for forensic investigations as well as legal systems.

## Materials & Methods:

This is an in vivo and comparative study including a sample size 120 patients visiting the Dental College and Hospital in the Department of Oral and Maxillofacial Pathology. The Institutional Ethics Committee of the University gave its approval for conducting the study (NIMSUR/IEC/2022/297). Participants consent was obtained before the study. The study recruited participants aged ≥18 years with permanent maxillary canine teeth that had fully erupted with a fully formed shape. Exclusion criteria included patients who had dental pathologies that impacted the dimensions of the tooth crown or surface area of the tooth, as well as patients with alignment or prosthetic restoration problems such as malalignments. Any rotten or structurally compromised teeth, including carious, periodontitis, or periapical lesion-affected teeth were not included in the sample.

## Study design:

The study used orthopantomographs (OPG) and intraoral periapical radiographs (IOPA) for digital analysis. All the necessary precautions to minimize radiation exposure were followed and this includes wearing lead aprons and thyroid collars while taking the orthopantomographs (OPG) and the intraoral peripheral radiographs (IOPA). IOPA radiographs were taken in paralleling/long cone technique in order to get least distortion and constant magnification. The radiographic images were obtained in JPEG format to ease subsequent digital analysis by AutoCAD software, where the actual measurements of the teeth as well as the pulp chamber were accurately determined. The ratio of the pulp to tooth was determined from every radiograph and is important when comparing the age estimation using regression models.

## Formula for age estimation:

Regression models based on the pulp/tooth area ratio were used to estimate age using the following formula:

Age=Intercept + (Pulp/Tooth Ratio) x (Coefficient).

Here, the pulp/tooth ratio is the ratio of pulp chamber size to tooth dimensions; the intercept takes the lowest value and the coefficient is the regression coefficient that is suitable for the given model.

## Data collection and analysis:

Each radiograph underwent meticulous measurements by a single observer using AutoCAD. Data on tooth and pulp areas were tabulated for statistical analysis using SPSS version 16. Descriptive statistics were generated using analysis of variance (ANOVA), while estimated age from digital measurements was tested for inter and intra observer reliability using intraclass correlation coefficients (ICC) calculated via SPSS version 16. Intraclass correlation coefficients (ICC) were calculated to assess reliability between predicted and actual ages, alongside gender-specific morphological variables.

## Results:

The gender distribution of the study sample, as shown in Table 1, indicates an equal representation of male and female participants, with 60 individuals (50% each) in both categories and a total sample size of 120 as seen in Table 1. For actual age, both OPG and IOPA groups, each consisting of 60 individuals, exhibited similar mean ages of 34.18 years, with standard deviations of 9.31 and standard error means of 1.20. No statistically significant difference was observed between the actual ages of the two groups (p=1.0). The OPG group (N=60) had an estimated mean age of 34.58 years, with a standard error mean of 1.46 and a standard deviation of 11.31. The mean estimated age difference between the OPG and IOPA groups has shown a statistical significance difference at p<0.05. On the other hand, the mean estimated age of the IOPA group (N=60) was 34.05 years, with a standard error mean of 1.37 and a standard deviation of 10.643.

The statistically significant difference in estimated ages favoring the IOPA group indicates that, although there were no significant differences in actual ages between the OPG and IOPA groups, the IOPA group provided a more accurate estimation of age than the OPG group, as seen in Table 2. The Table 3 illustrates an intergroup comparison of the mean change in actual and estimated ages within the IOPA (Intraoral Periapical Radiograph) group among males and females. Among males (N=30) in the IOPA group, the mean change in estimated age was 1.80 years, with a standard deviation of 1.297 and a standard error mean of 0.237. The mean difference in the change of estimated age between males and females was -1.00 years, with a t value of 0.263. A statistical significance difference has observed at p=0.01, suggesting that the change in estimated age for males within the IOPA group significantly differs from females. For females (N=30) in the IOPA group, the mean change in estimated age was 2.80 years, with a standard deviation of 1.627 and a standard error mean of 0.297. No statistically significant difference was observed in the change of estimated age for females within the IOPA group. Table 4 presents an intergroup comparison of the mean change in actual and estimated ages within the studied population between the OPG (Orthopantomogram) and IOPA (Intraoral Periapical Radiograph) groups. In the OPG group (N=60), the mean change in estimated age was 2.77 years, with a standard deviation of 1.079 and a standard error mean of 0.139. In contrast, the mean change in estimated age for the IOPA group (N=60) was 2.30 years, with a standard deviation of 1.544 and a standard error mean of 0.199.The mean difference in the change of estimated age between the OPG and IOPA groups was 0.467 years, with a t value of 1.919, observing a statistical significance difference at p=0.001. This suggests that the mean change in estimated age differs significantly between the OPG and IOPA groups, indicating potential variations in the effectiveness of these imaging techniques in estimating age within the studied population. Table 5 describes the results of a regression analysis conducted for the overall studied subjects encompassing both the IOPA (Intraoral Periapical Radiograph) and OPG (Orthopantomogram) groups. The coefficient of determination (R-squared) for the regression model is 0.947, indicating that approximately 94.7% of the variability in the dependent variable (presumably estimated age) can be explained by the independent variables included in the model. The adjusted R-squared value (adjusted for the number of predictors in the model) is also high at 0.946. These findings clearly imply that the method of regression yields a very precise forecast. Table 6 provides the overall descriptive statistics of the pulp/tooth ratio under the observed population, encompassing both the OPG (Orthopantomogram) and IOPA (Intraoral Periapical Radiograph) groups. For the OPG group (N=60), the mean pulp/tooth ratio is 0.298, with a standard deviation of 0.245 and a standard error mean of 0.031. The mean pulp/tooth ratio for the IOPA group (N = 60) is 0.289, with a standard deviation of 0.236 and a standard error mean of 0.030. This is a small decrease from the norm. These descriptive statistics of estimated age for all the studied subjects provide the necessary information to characterize the overall distribution and variance of the pulp/tooth ratio in the population under investigation for both imaging modalities, with the IOPA group anticipated to have a significant impact on the model. They speculate that there may be a small difference in the mean pulp/tooth ratio between the two groups, with the OPG group having a marginally higher mean ratio than the IOPA group. Thus, based on these findings it has been observed that IOPA is a more accurate method of estimating age than OPG.

## Discussion:

Personal identification serves as a critical foundation in legal medicine, criminal investigation, genetic research and disaster victim identification [11, 1]. Within the forensic sciences, forensic dentistry or forensic odontology plays a vital role by leveraging dental expertise to support criminal and civil investigations within the criminal justice system [12]. Modern forensic odontology, as outlined by Keiser-Nielsen and Bosmans, encompasses three primary investigative domains: assessment of injuries to the jaws, oral tissues and teeth analysis of bite marks for identification purposes; and examination of dental remains, including restorations, for identification and exclusion [12, 13-14]. Techniques such as morphological, biochemical and radiological methods are employed depending on the nature of the investigation [2]. Anthropometry, fingerprint analysis, sex determination, age estimation, height measurement, individual identification and blood group analysis are examples of traditional techniques for personal identification [3]. Age estimation holds significant importance in various forensic, clinical, legal and social contexts, aiding in responsibilities, treatment planning and legal proceedings [15, 16]. Teeth are among the most reliable indicators of age, especially during developmental phases in the early and second decades of life, owing to their predictable growth patterns [16]. Radiographic techniques offer a non-invasive, repeatable method for age estimation, applicable in forensic and archaeological contexts for both known and unknown individuals [17]. Gustafson's pioneering work on radiographic changes in dental structures remains foundational in forensic age estimation, widely adopted by pathologists and odontologists [18- 19]. The pulp-tooth area ratio method, introduced by Cameriere *et al.* has demonstrated superior accuracy in adult age estimation compared to traditional methods [20, 21-22]. This method calculates the ratio of pulp chamber area to tooth area using radiographs, with studies suggesting that bicuspids provide better estimates than canines [22, 23]. Radiographic techniques, whether using intraoral periapical or panoramic methods, have been instrumental in assessing skeletal maturity and secondary dentin deposition, both of which correlate closely with age [20, 24- 25].

The methods used in this investigation were identical to those of Bosmans *et al.* [14] and had found a strong correlation (correlation coefficient = 0.97) between age estimates derived from IOPA and OPG, affirming the reliability of the pulp-tooth area ratio for age estimation [17]. The present findings support previous research indicating that the breadth of the pulp chamber serves as a reliable age indicator, underscoring the utility of radiographic techniques in forensic age estimation. In order to validate the accuracy of radiographic methods in forensic applications, studies by Cameriere *et al.* and Bosmans *et al.* have also established that there is no statistically significant difference between estimated and chronological ages [14, 17]. Statistical analysis in the present study demonstrated a significant regression model (p < 0.001), indicating that variables such as pulp-tooth area ratio from IOPA and OPG collectively influence age estimation accuracy across our study population. They also stated some limitations that exist in age estimation methods, such as the pulp chamber size differences resulting from the growth process or ethnicity differences and as such require period-specific validation studies. Furthermore, though techniques based on radiographic images present evident advantages in terms of invasiveness and reproducibility, a disparity with practical results could be observed due to the existence of factors such as tooth wear, attrition and variations in the shape of the pulp chamber [17- 18, 26]. Possible research avenues for future investigations may therefore include increasing sample size and using more than one tooth for age estimation in order to increase the validity of the results. Further investigations on models for age estimation should be carried out on a population-specific basis owing to genetic differences as well as the influence of the environment on dental maturity.

## Conclusion:

In conclusion, radiographic methods using intraoral periapical and panoramic radiographic pulp-tooth area ratios give reliable estimates of age in forensic as well as clinical situations. Thus the present research ties to the rich literature for bolstering these techniques, pointing to their relevance in legal medicine and forensic sciences. More developments in imaging methods and mathematical algorithms call for enhanced precision in age estimation, which will be of great benefit in both forensic and clinical settings all over the world.

## Figures and Tables

**Table 1:** Demographic details

**Gender**	**Number (%)**
Male	60(50%)
Female	60(50%)
Total	120

**Table 2:** Comparative difference of changes observed in opg and iopa between actual age and estimated age

	**Group**	**N**	**Mean**	**Std. Deviation**	**Std. Error Mean**	**Mean diff**	**t value**	**p value**
ACTUAL AGE	OPG	60	34.18	9.316	1.2	0	0	1.0**
	IOPA	60	34.18	9.316	1.2			
ESTIMATED AGE	OPG	60	34.58	11.313	1.46	0.53	1.54	0.05*
	IOPA	60	35.05	10.643	1.37			

**Table 3:** Intergroup comparison of mean change of actual and estimated age of IOPA group among males and females

**Group IOPA**	**N**	**Mean**	**Std. Deviation**	**Std. Error Mean**	**Mean diff**	**T value**	**p value**
Male	30	1.8	1.297	0.237	-1	0.263	0.01*
Female	30	2.8	1.627	0.297			
*statistically significant
**statistically non-significant

**Table 4:** Intergroup comparison of mean change of actual and estimated age of studied population among OPG and IOPA groups

**Group**	**N**	**Mean**	**Std. Deviation**	**Std. Error Mean**	**Mean diff**	**t value**	**p value**
OPG	60	2.77	1.079	0.139	0.467	1.919	0.001*
IOPA	60	2.3	1.544	0.199			
*statistically significant
**statistically non-significant

**Table 5:** Regression analysis for overall studied subjects of both IOPA and OPG group

**R**	**R Squarer^2^**	**Adjusted R Square AR^2^**	**Std. Error of the Estimate (SEE)**	**p value**
.973a	0.947	0.946	2.155	0.001*
*Statistically significant
**statistically non-significant

**Table 6:** Overall Descriptive statistics of pulp/tooth ratio in studied population among OPG and IOPA groups

**R**	**R Square r^2^**	**Adjusted R Square AR^2^**	**Std. Error of the Estimate (SEE)**	**p value**
.973a	0.947	0.946	2.155	0.001*
